# Sex differences in FASN protein concentrations in urinary exosomes related to serum triglycerides levels in healthy adults

**DOI:** 10.1186/s12944-023-01936-7

**Published:** 2023-10-19

**Authors:** Tao Li, Wen Meng, Tian Ci Liu, Yi Zhao Wang, Man Zhang

**Affiliations:** 1 Clinical Laboratory Medicine, Beijing Shijitan Hospital, Capital Medical University, Beijing, 100038 China; 2 Beijing Key Laboratory of Urinary Cellular Molecular Diagnostics, Beijing, 100038 China; 3 Peking University People’s Hospital, Beijing, 100044 China; 4 Institute of Regenerative Medicine and Laboratory Technology Innovation, Qingdao University, Qingdao, 266071 China

**Keywords:** Urinary exosomes, Fatty acid synthesis, FASN, Sex differences, Triglyceride

## Abstract

**Background:**

Dysregulation of lipid metabolism is the most prominent metabolic alteration observed in obesity, cancer, and cardiovascular diseases. The present study aimed to explore the sex differences associated with lipid metabolism in urinary exosome proteins, and evaluate the correlation of urinary exosome proteins with serum lipid biomarkers.

**Methods:**

The key enzymes regulating lipid metabolism in healthy adults were screened using urinary exosome data. Urinary exosomes were isolated from 120 healthy subjects and the expression of urinary proteins was assessed by Western blotting and *ELISA*. The correlation between urinary protein concentrations and the levels of serum lipid biomarkers was analyzed using correlation analysis.

**Results:**

Three urinary exosome proteins, namely fatty acid synthase (FASN), phosphoenolpyruvate carboxykinase (PCK1), and ATP-citrate synthase (ACLY) were identified, and only FASN showed sex differences. Sex differences were also observed in the serum triglyceride (TG) levels. Healthy males had higher FASN levels than females, and a moderate positive correlation was found between FASN concentrations and serum TG levels in healthy males (*r* = 0.479, *P* < 0.05). FASN concentrations in different age groups were positively correlated with the level of serum TG (18 ~ 30 years, *r* = 0.502; 31 ~ 44 years, *r* = 0.587; 45 ~ 59 years, *r* = 0.654; all* P* < 0.05). In addition, FASN concentrations was positively related to the increase in serum TG levels (range:1.0 ~ 1.7 mmol/L; *r* = 0.574, *P* < 0.05).

**Conclusions:**

Sex differences were observed in urinary exosome FASN protein levels in healthy adults. FASN protein levels positively correlated with increased serum TG levels. FASN may serve as a novel biomarker to evaluate fatty acid synthesis in the human body.

**Supplementary Information:**

The online version contains supplementary material available at 10.1186/s12944-023-01936-7.

## Background

Lipids are water-insoluble substances, that include phospholipids, sterols, sphingolipids, terpenes, and fatty acids. Lipids based on their constituent molecules can be classified as simple or complex. Simple lipids can be described as those that yield at most two types of products during hydrolysis. Complex lipids are those that produce three or more products after hydrolysis [1, 2]. In addition to providing sufficient energy, lipids are extensively spread in the organelles of cells and involved in various signal transduction pathways [3, 4]. It is commonly known that women generally have a higher body fat content than men [5]. Sex differences in fat metabolism are influenced by multiple factors [6]. Dysregulation of lipid metabolism, such as elevated serum triglycerides (TG) and cholesterol levels, greatly increases the incidence of cardiovascular diseases and diabetes [7, 8]. Furthermore, numerous studies have demonstrated that lipid metabolism disorders are the most prominent metabolic alteration in cancers, especially breast and cervical cancers [9, 10]. Cancer cells harness lipid metabolism to obtain energy to alter the tumor microenvironment, thereby promoting the survival, proliferation, invasion, and migration of cancer cells. Since homeostasis of lipid metabolism is essential for cancer development, inhibitors of rate-limiting enzymes are promising targets for treatment [11]. Numerous preclinical studies investigating potential inhibitors of a variety of lipid metabolism enzymes for cancer treatment, including inhibitors of cholesterol synthesis [12], statin family drugs [13], and inhibitors of fatty acid synthesis [14, 15], have been reported. The early and timely detection of lipid metabolism disorders is essential for human health.

Urine is the second most commonly used biological fluid in clinical diagnosis, and commonly contains epithelial and blood cells, bacteria, viruses, and exosomes [16]. Urinary exosomes are small circular membranous vesicles with a diameter of 30 ~ 150 nm derived by kidney epithelial cells or urinary tract cells, reflecting the physiological and pathological state of the body in health and diseases [17, 18]. Numerous studies have pointed out that urinary exosomes can serve as a source of novel biomarkers reflecting the physiological and pathophysiological state of the human body [19, 20]. However, while representing noninvasively collected and easily prepared biological samples, the potential role of urinary exosomes in reflecting lipid metabolism in *vivo* and the correlation of urinary exosome biomarkers with serum lipid metabolism markers remain unknown. Therefore, we hypothesized that biomarkers are present in urinary exosomes and capable of reflecting the status of lipid metabolism.

In this study, rate-limiting enzymes related to the regulation of lipid metabolism, including fatty acid and cholesterol metabolisms were screened using urinary exosome data from healthy subjects. In addition, the expression levels of key rate-limiting enzymes were assessed, and their correlation with serum lipid metabolism biomarkers levels was also evaluated.

## Methods

### Study cohort

Healthy subjects who were first admitted for physical examination at Beijing Shijitan Hospital, were sampled between January 2021 and January 2022. The inclusion criteria for the healthy subjects were as follows: (1) Physical examination showed no tumors, autoimmune diseases, diabetes, cardiovascular diseases, and chronic liver disease; (2) Laboratory test results were within normal ranges; (3) No acute urinary tract infections were observed for healthy subjects; (4) No history of long-term drug use, such as hypolipidemic drugs and glucocorticosteroids. Clinical characteristics were obtained using electronic medical records, such as height, weight, drinking history, smoking history, and diabetic family history.

### Ethics approval and consent to participate

The present study was approved by the Medical Ethics Committee of Beijing Shijitan Hospital (No. sjtkyll-1x-2021(115)) and followed by the principles of the Declaration of Helsinki. Written consent of all healthy subjects was obtained before being included in this study.

### Serum biomarkers measurement

Serum samples were obtained from the peripheral veins of healthy subjects at the state of fasting and then centrifuged at a speed of 1500 rpm for 10 min. Serum lipid biomarkers were measured using BECKMAN COULTER AU5800 instrument (Brea, California, USA). Complete blood counts were measured using SYSMEX, XE-5000 (Kobe, Japan). Serum tumor markers levels, including alpha fetoprotein (AFP), carcinoembryonic antigen (CEA), carbohydrate antigen 199 (CA199), and prostate specific antigen (PSA) were measured using the electrochemiluminescence method (Roche Cobas E601, Basel, Switzerland). HbA1c levels were also analyzed in our laboratory (TOSOH G8, Yamaguchi, Japan).

### Urine collection and extraction of urinary exosomes

Thirty mL of first morning urinary samples were obtained from the healthy subjects. Each sample were centrifuged at a speed of 1500 g for 10 min, followed by removing the cells and debris at 10000 g × 30 min. Concentrate processed urine to 10 mL using ultrafiltration tubes. Urinary exosomes were extracted using 35-nm qEV10 iZON size-exclusion chromatography (SEC) columns (H-wayen Biotechnologies, Shanghai, China). The obtained urinary exosome samples were stored at a temperature of -80 °C until usage for further study.

### Identification of urine derived exosomes

For transmission electron microscopy (TEM), 10 µl of urinary exosome sample were placed on a dry copper mesh for 10 min. A drop of 2% uranyl acetate was added onto the urinary exosome sample for 1 ~ 3 min. After drying for 10 ~ 15 min, morphology of exosomes under the microscope was acquired (JEM-1200 EX, Tokyo, Japan).

For nanoparticle tracking analysis (NTA), each sample was diluted to an appropriate concentration and directly tested for exosome particle size (ZETA VIEW, PARTICLE METRIX, HAMBURG, GERMANY). A typical result of NTA is shown in Supplementary Materials 1.

### Mass spectrometry (MS)

**Fig. 1:**
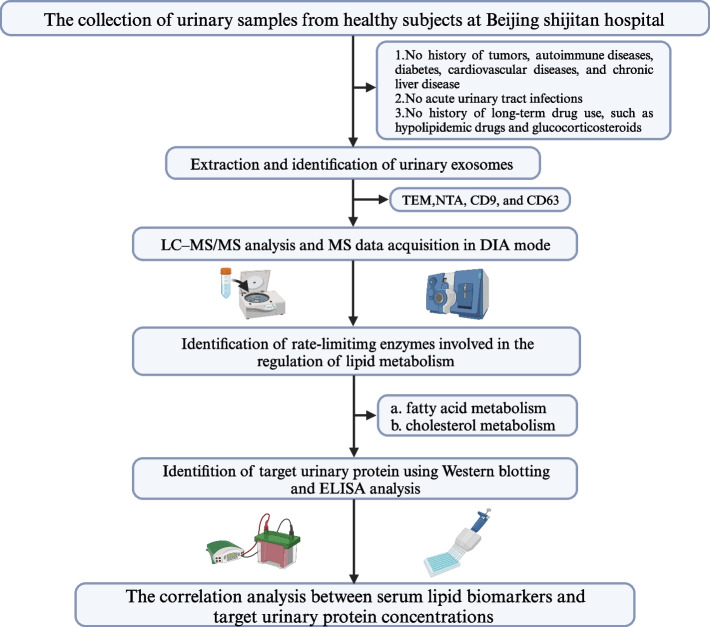
The detailed workflow of this study

MS proteomic data of enzymes involved on the regulation of lipid metabolism were collected from healthy subjects (Females: C1 ~ F1 and Males: C2 ~ F2; age range: 18 ~ 79 years). This study focused on the rate-limiting enzymes related to the regulation of lipid metabolism, including fatty acid and cholesterol metabolisms. Simplified workflow of the MS analysis is briefly described as follows. 1 ug of the sample was injected and the liquid quality was tested. Mass spectra were analyzed by an ORBITRAP ECLIPSE mass spectrometer using data-independent acquisition (DIA) mode. The results of MS were obtained in the human database (www.UniProt.org). When fold change > 1.5 and *P* value < 0.05, it was considered to suggest the presence of differentially expressed. Cluster heat map and primary component analysis (PCA) were produced and visualized using R 4.0.1 version (USA). A detailed workflow of this study is summarized in Fig. 1 (Created with BioRender.com).

### Western blotting analysis

The study randomly selected 12 males and 12 females among the 120 healthy subjects. Samples were lysed in RIPA lysis buffer for 30 min. The total urine protein level was measured by BCA Protein Assay Kit (Solarbio, Beijing, China) and selected sample was loaded onto 8% ~ 15% sodium dodecyl sulfate–polyacrylamide gel. Electrophoresis was performed at 60 V to 120 V, followed by transfer to the membrane (Bio-Rad, Hercules, USA). A 5% blocking solution was added, and placed on a rocker for 2 h. Anti-CD9 (Abcam, ab236630), anti-CD63 (Abcam, ab271286), and anti-fatty acid synthase (Proteintech, 10624–2-AP) antibodies were used in suitable ratio of 1:1000 ~ 3000, and then overnight incubation at a temperature of 4 °C. Next day, washing three times, secondary antibody was added at a ratio of 1:3000 for 2 h. After rewashed three times, enhanced chemiluminescence (ECL) solution (Bio-Rad, cat. **#**170–5061, California, USA) was added for exposure. Raw Western blot images are shown in Supplementary Materials 2.

### ELISA analysis of urinary exosome FASN protein level

Samples were lysed in RIPA lysis buffer for 30 min. Total protein was measured using BECKMAN COULTER AU5800 instrument (Brea, California, USA). Concentrations of urinary protein were measured using an *ELISA* kit (Shanghai Enzyme-linked Biotechnology Co., Ltd; lot:202204, Catalog number: YJ84850, Wuhan, China). Based on the instruction of manufacturer, 50 μl of samples were added to each well and then incubated for 30 min at a temperature of 37 °C. After three times of washing the plate, HRP-Conjugate reagent was added and incubated for 30 min. After washing, 50 μl of chromogen A and B solution were added to each well plate for 10 min at a temperature of 37 °C. Finally, adding stop solution for measuring absorbance. The value of optical density was measured at 450 nm (Thermo Fisher Scientific, California, USA), with each sample tested three times. The FASN concentrations was calculated based on the standard curve.

### Statistical analysis

For normally distributed data, the mean ± SD is presented and compared using Student’s t-test. For non-normally distributed continuous variables, Mann–Whitney test were used to analysis. The relationships between the levels of serum lipid metabolism biomarkers and urinary protein concentrations were analyzed by Pearson’s correlation or Spearman’s rank coefficient. The analysis of all variables was analyzed by GraphPad Prism software (California, USA). Differences in analysis were considered to be significant if *P* < 0.05*.*

## Results

### Key lipid metabolism enzymes in urinary exosomes

**Fig. 2:**
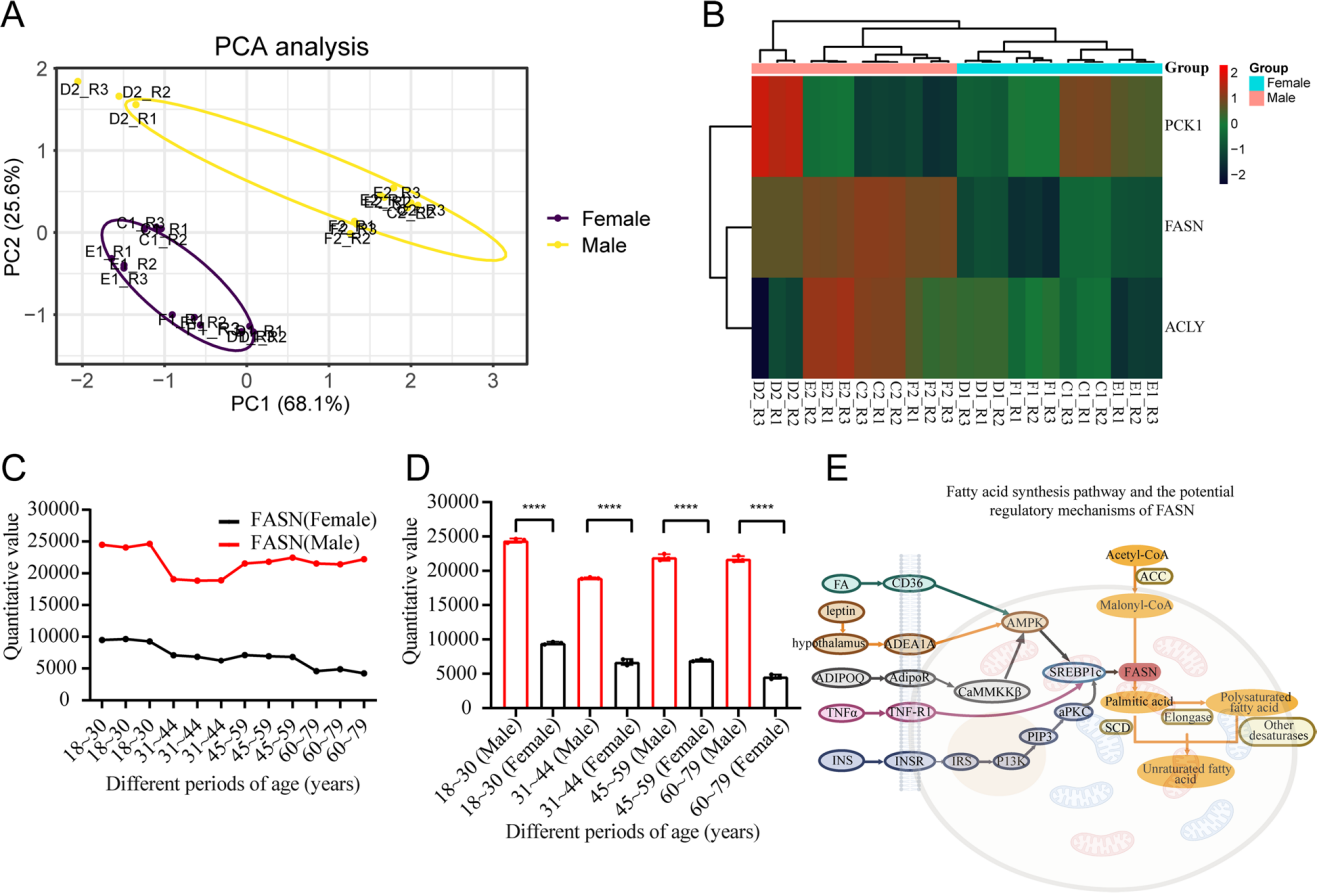
Key lipid metabolism enzymes in urinary exosomes. **A** The result of PCA analysis, colored by groups, and each ellipse plot represents the CI of 95% confidence coefficient for individual groups (Female: C1 ~ F1; Male: C2 ~ F2). **B** Hierarchical clustering heatmap analysis of proteins in healthy female and male groups (Female: C1 ~ F1; Male: C2 ~ F2; age range:18 ~ 79 years). Red represents upregulated and green represents downregulated. **C** The trend of FASN abundance in different age groups. **D** The differences of FASN abundance in different age groups. **E** Classical fatty acid synthesis pathway and the potential regulatory mechanisms of FASN. (ns: no significance; *:*P* < 0.05; **P < 0.01; ***: *P* < 0.001)

This study identified three proteins associated with lipid metabolism in urinary exosomes, namely, FASN, ATP-citrate synthase (ACLY), and phosphoenolpyruvate carboxykinase (PCK1). PCA showed that the data for the three urine proteins were reliable (Fig. 2A). The clustered heatmap showed a distinction between ages groups for the three urine proteins (Fig. 2B). The trends of FASN abundance demonstrated that healthy males had higher levels than females in different age groups (Fig. 2C). Sex differences in different age groups for the quantitative value of the urine FASN protein were observed (all *P* < 0.001; Fig. 2D). However, the urine ACLY and PCK1 proteins did not show these trends in sex differences (Supplementary Materials 3). The classical fatty acid synthesis pathway and potential regulatory mechanisms of FASN are summarized in Fig. 2E [21].

### Clinical characteristics of healthy subjects

**Fig. 3:**
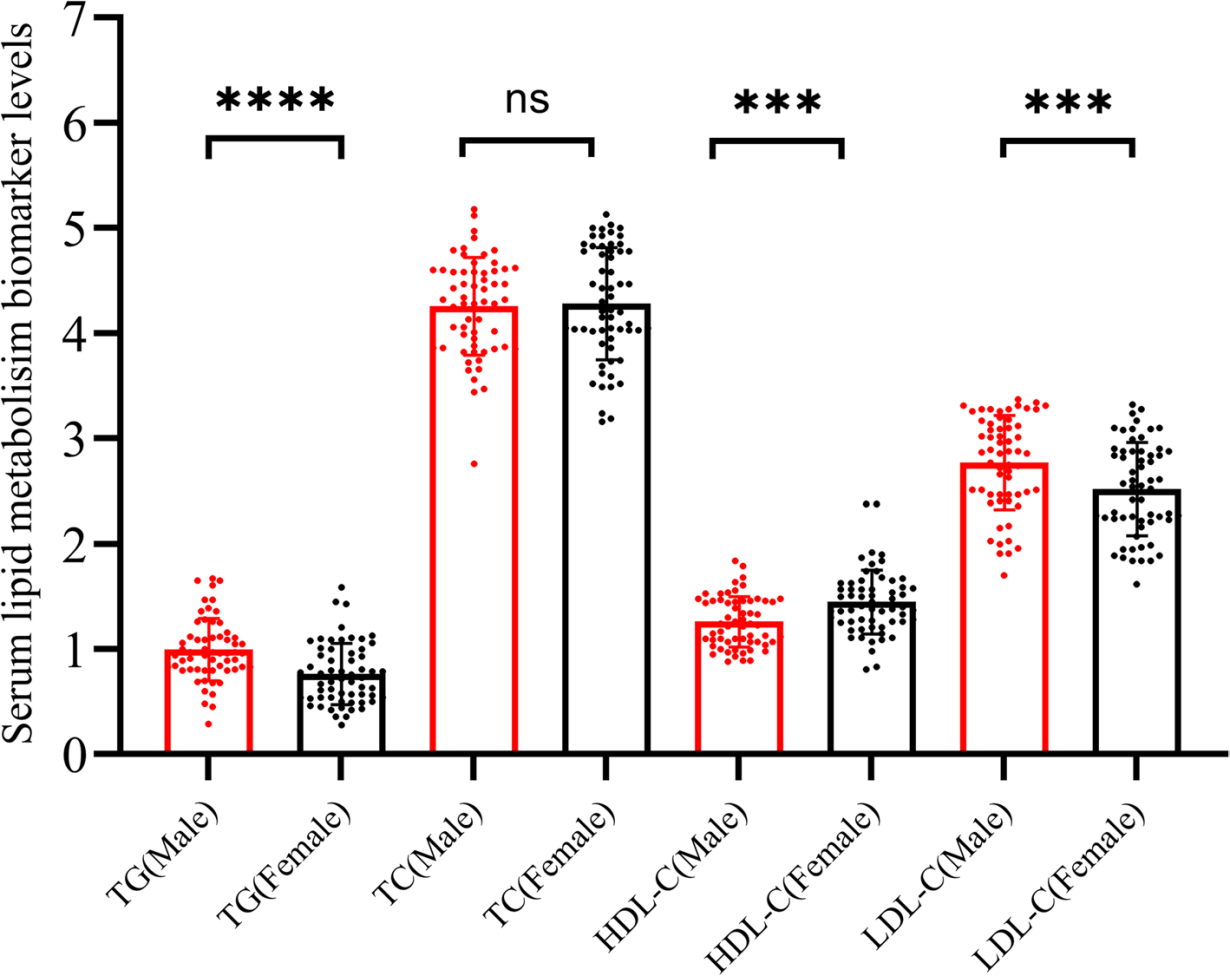
Sex differences in serum lipid metabolism biomarkers. Healthy males had higher serum TG and HDL-C levels. A lower level of LDL-C than females was observed. Sex difference in serum total cholesterol levels was not observed (ns: no significance; *:*P* < 0.05; **P < 0.01; ***: *P* < 0.001)

Healthy subjects (*n* = 120) were included in the validation set. The clinical data showed that no difference in age between males and females. Furthermore, healthy males had a higher level of red blood cell (RBC) counts, aspartate aminotransferase (ALT), alanine aminotransferase (AST), albumin (ALB), and serum creatinine (Cr) levels, and lower levels of estimated glomerular filtration rate (eGFR) was observed (*P* < 0.05; Table 1). Interestingly, sex differences were also observed in the level of serum TG, HDL-C, LDL-C (all *P* < 0.01; Fig. 3). Sex differences were not observed in other clinical features.

**Table 1:** Clinical characteristics of healthy subjects

**Variables**	**Male (*n* = 60)**	**Female (*n* = 60)**	***P* value**
Age, years	37.62 ± 10.64	37.25 ± 9.94	ns
BMI, kg/m^2^	22.46 ± 3.01	22.02 ± 2.53	ns
Smoking history, yes, (n, %)	8 (13.33%)	3 (5.00%)	ns
Drinking history, yes, (n, %)	5 (8.33%)	1 (1.67%)	ns
Diabetic family history, yes, (n, %)	9 (15.00%)	12 (20.00%)	ns
WBC counts × 10^9^/L	5.78 ± 1.73	5.52 ± 1.14	ns
RBC counts × 10^12^/L	5.08 ± 0.33	4.48 ± 0.27	***
Hb, g/L	155.28 ± 8.49	156.55 ± 7.11	ns
PLT counts × 10^9^/L	230.92 ± 47.01	248.68 ± 52.84	ns
AST, U/L	19.35 ± 3.76	16.93 ± 3.55	**
ALT, U/L	20.45 ± 6.40	14.72 ± 5.24	**
ALB, g/L	44.50 ± 1.91	43.46 ± 1.75	*
FBG, mmol/L	5.15 ± 0.41	5.36 ± 0.36	ns
HbA1c, %	5.41 ± 0.31	5.42 ± 0.37	ns
Serum Cr, μmol/L	69.53 ± 5.16	58.53 ± 6.09	***
eGFR, mL/min/1.73m^2^	104.65 ± 10.28	106.00 ± 8.97	**
Urine protein, negative, (n, %)	60 (100)	60 (100)	ns
CEA, ng/mL	1.75 ± 0.75	2.62 ± 0.56	ns
PSA, ng/mL	0.91 ± 0.49	NA	NA
CA199, U/mL	9.19 ± 7.41	8.63 ± 7.64	ns
AFP, ng/mL	3.07 ± 1.41	2.75 ± 1.29	ns

*BMI* Body Mass Index, *WBC* White blood cell, *RBC* Red blood cell, *Hb* Hemoglobin, *PLT* Platelet, *AST* Aspartate aminotransferase, *ALT* Alanine aminotransferase, *ALB* Albumin, *FBG* Fasting Blood Glucose, *Cr* Creatinine, *eGFR* Estimated glomerular filtration rate, *CEA* Carcinoembryonic antigen, *PSA* Prostate Specific Antigen, *CA 199* Carbohydrate antigen 199, *AFP* Alpha Fetoprotein

*P* value: ns, no significance; *, *P* < 0.05; **, *P* < 0.01; ***, *P* < 0.001

### FASN evaluation by Western blot and ELISA analysis

**Fig. 4:**
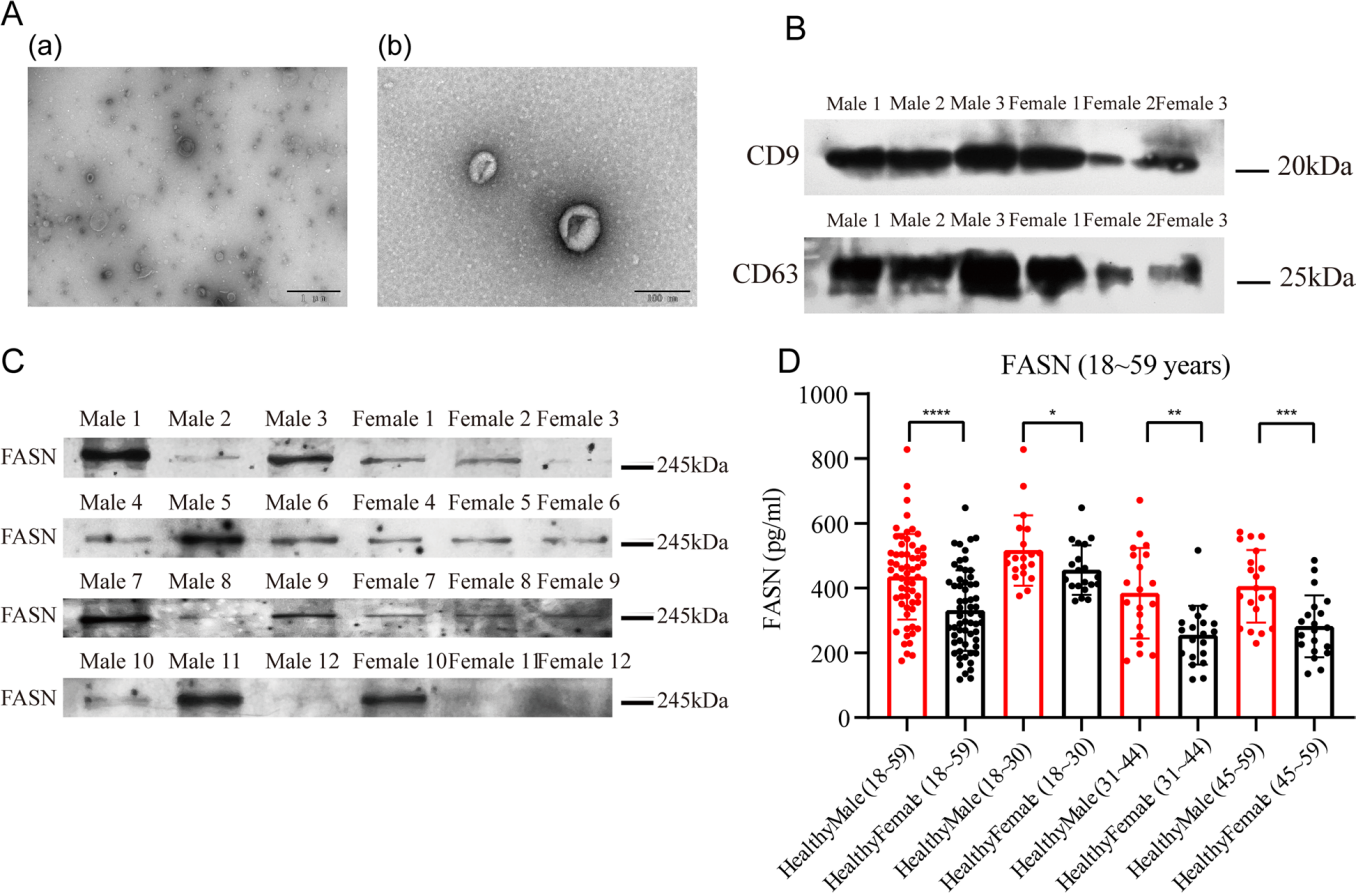
Identification of FASN by Western blotting and *ELISA* analysis. **A** Representative TEM images of urinary exosomes (scale bars; a = 1um, b = 100 nm). **B** Western blot images of urinary exosome markers CD9, CD63. **C** Western blot images of urinary exosome FASN protein (Females: *n* = 12; Males: *n* = 12). **D** Sex difference was observed in different age groups. (ns: no significance; *:*P* < 0.05; **P < 0.01; ***: *P* < 0.001)

Representative TEM images of urinary exosomes are shown in Fig. 4A (scale bars; a = 1 µm, b = 100 nm). Urinary exosome markers, including CD9 and CD63, were identified using Western blotting (Fig. 4B). According to the NTA measurements, almost all urinary exosomes are 123.7 nm in size (Supplementary Materials 1). The expression level of FASN was determined by Western blotting. Compared with healthy females, in healthy males there was a trend towards increased levels of urine FASN protein (Fig. 4C). Finally, the concentration of the urine FASN protein was analyzed in 120 healthy subjects using *ELISA*. Significant sex differences were observed in the different age groups (18 ~ 30 years, *P* = 0.047; 31 ~ 44 years, *P* = 0.0013; 45 ~ 59 years, *P* = 0.0006; Fig. 4D).

### Correlation between urinary protein FASN concentrations and serum TG levels

**Fig. 5:**
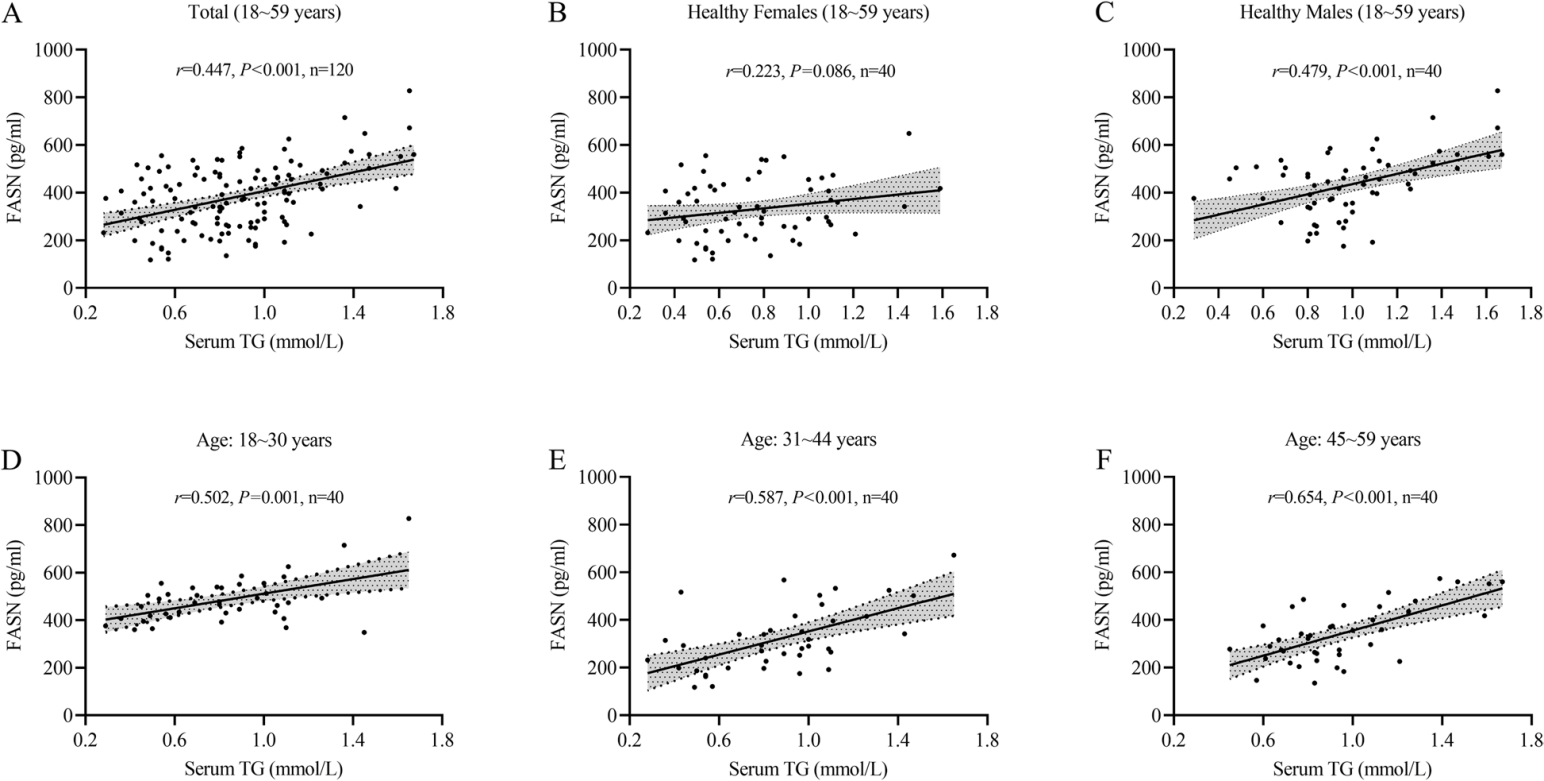
Correlation between serum TG levels and the concentrations of urinary protein FASN. **A** A positive correlation between serum TG levels and FASN concentrations (*n* = 120; *r* = 0.447; *P* < 0.001). **B** No correlation was observed in healthy females (*r* = 0.223; *P* = 0.086). **C** FASN concentrations were positively correlated with serum TG levels in healthy males (*r* = 0.479; *n* = 40; *P* < 0.0001). **D**, **E**, and **F** A positive correlation was observed between serum TG levels and FASN concentrations in different age groups (18 ~ 30 years, *r* = 0.502; 31 ~ 44 years, *r* = 0.587, 45 ~ 59 years; *r* = 0.654; all* P* < 0.05). (ns: no significance; *:*P* < 0.05; **P < 0.01; ***: *P* < 0.001)

In healthy participants, a positive correlation was found between FASN concentrations and the level of serum TG (*r* = 0.447, *P* < 0.05; Fig. 5A). FASN concentrations had a positive correlation with serum TG levels in healthy males (*r* = 0.479, *P* < 0.05; Fig. 5C). However, this correlation was not observed in healthy females (*r* = 0.223, *P* = 0.086; Fig. 5B).

Meanwhile, FASN concentrations positively correlated with the level of serum TG in different age groups (18 ~ 30 years, *r* = 0.502; 31 ~ 44 years, *r* = 0.587; 45 ~ 59 years, *r* = 0.654; all* P* < 0.05; Fig. 5D, E, and F).

### Correlation between stratified serum TG levels and urinary protein FASN concentrations

**Fig. 6:**
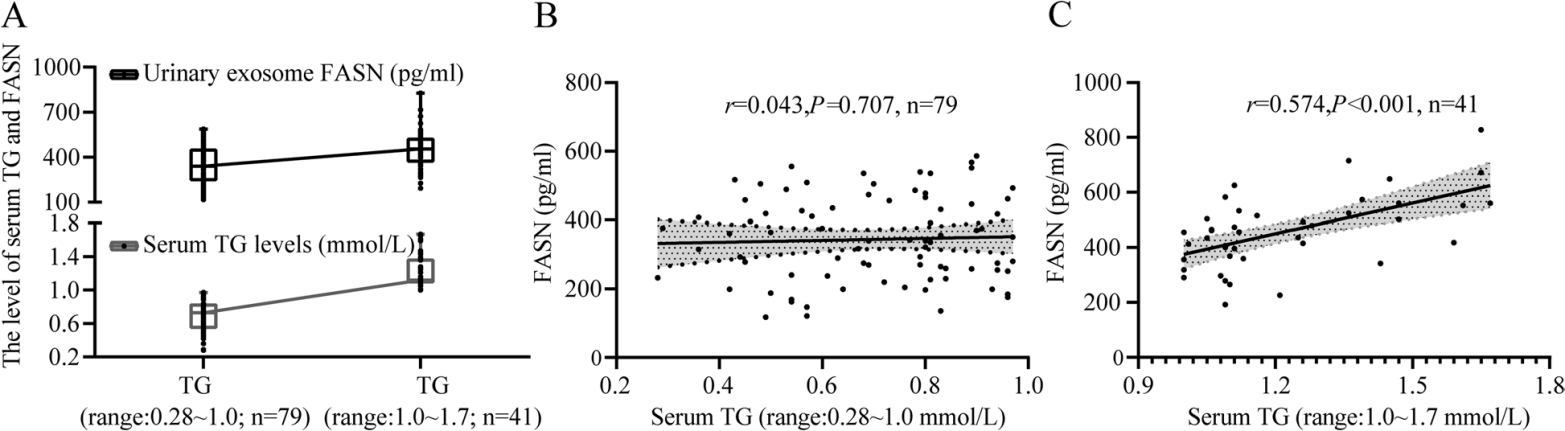
Correlation between stratified serum TG levels and urinary protein FASN concentrations. **A** The trend of FASN concentrations was consistent with serum TG levels. **B** No correlation was observed between serum TG levels (range: 0.2 ~ 1.0 mmol/L) and FASN concentrations (*n* = 79; *r* = 0.043, *P* = 0.707). **C** A positive correlation was found between serum TG levels (range: 1.0 ~ 1.7 mmol/L) and FASN concentrations (*n* = 41; *r* = 0.574, *P* < 0.001)

Because the serum TG levels were within normal range, we further divided these healthy subjects into two groups, including low (*n* = 79; TG range: 0.2 ~ 1 mmol/L) and high TG groups (*n* = 41; TG range: 1.0 ~ 1.7 mmol/L). Interestingly, the trend shown by FASN concentrations was consistent with that shown by serum TG levels (Fig. 6A). FASN concentrations positively correlated with high serum TG levels (*r* = 0.574, *P* < 0.0001; Fig. 6C). However, positive correlation was not observed in the low serum TG levels group (*r* = 0.043, *P* = 0.707; Fig. 6B).

## Discussion

According to the global burden of disease study, cardiovascular diseases, diabetes, cancer, and obesity are the most common diseases that threaten human health [22–24]. Homeostasis of the lipid metabolism, particularly fatty acid synthesis, is crucial for maintaining the functional status of the human body. Fatty acids are the main components of triglycerides, phospholipids, and glycolipids, and provide sufficient energy to support the activities of organisms. Although serum TG level can be used to evaluate overall fatty acid synthesis in the body, it may not serve as an early biomarker for predicting lipid metabolism disorders [25, 26]. Urinary exosomes are emerging sources of biomarkers widely implicated in diseases onset and progression [19]. In this study, FASN, ALCY, and PCK1 proteins were identified in the urinary exosomes of healthy adults. Differences in FASN but not in ACLY and PCK1 proteins concentrations were observed across the sex and age groups. Although serum biomarkers levels were within the normal range, healthy males had higher level of RBC counts, AST, ALT ALB, Cr, and TG than healthy females. However, healthy males had lower level of eGFR than healthy females. Positive correlations were found between urinary protein FASN concentrations, RBC counts, and ALT levels (Supplementary Materials 4). A recent study has shown that the excretion rate of urinary extracellular vesicles decreases with a decline in nephron mass [27]. In this study, no correlation was found between FASN concentrations and eGFR. The lack of correlation may be due to the normal renal function in healthy subjects.

The biological function of FASN is to transform excess carbohydrates into fatty acids, which can be esterified into triacylglycerols. Eventually, these stored or used for energy through β-oxidation [10, 28]. This study demonstrated the existence of the urinary exosome FASN protein, which may serve as a non-invasive and stable biomarker to evaluate fatty acid synthesis status. Furthermore, earlier studies have reported that serum TG levels gradually increase with age [29]. However, serum TG levels in males increase until 45 years and then slightly decrease, whereas TG levels continue to increase with age in females [30]. In this study, FASN concentrations were positively correlated with increased serum TG levels. Freedman et al. found a distinct difference in fat distribution between healthy males and females, and point out that this sex difference was related to differences in lipid levels [31]. Previous studies have demonstrated that females have developed specific mechanisms that facilitate the storage of adipose tissue, whereas males are able to more effectively mobilize stored fat [32, 33]. Differences in the oxidation of base fatty acids, modulation of lipolysis by catecholamines and insulin, and post-prandial fatty acid storage may promote sex differences in regional fat distribution [6]. Jenny and Karen have reported that basic genetic differences are ultimately determined by XX or XY sex chromosome complements between males and females [34].

Metabolism of is a complex and multifactorial process. Urine, an end product of the body’s metabolism, is filtered by the kidneys and excreted through the urinary tract. Urinary exosomes may be derived from urinary tract cells or kidney epithelial cells, reflecting the functional state of the human body. Based on these results, the urinary exosome FASN protein may be an end-product excreted by the kidneys that reflects the state of fatty acid metabolism in the body. Sex differences in FASN levels may be due to the beneficial effects of endogenous estrogen in females and different fat storage sites in men and women. A study is being conducted to identify the potential underlying processes for sex differences about FASN levels.

## Conclusions

This study explored the potential clinical applications of urinary exosome FASN protein in healthy adults. Urinary exosome FASN protein showed sex differences in healthy adult and had a positive correlation with increased serum TG levels. This study may provide a novel biomarker for the evaluation and monitoring of fatty acid synthesis, and further prevent the onset of the obesity, cardiovascular diseases, diabetes, and cancer.

### Limitations

This study has several limitations. First, due to differences in water consumption among healthy individuals, urine samples may contain a lower quantity of urinary exosomes than blood samples. In addition, the lack of serum urinary exosome data corresponding to healthy subjects, sex differences, and correlation between serum exosome FASN protein concentrations and serum TG levels were not evaluated. Finally, as this was a single-center cohort study, the sample size was not large, and the results require a larger sample size for further validation.

### Supplementary Information

**Additional file 1: Supplementary Materials 1.** The typical result of NTA analysis.

**Additional file 2: Supplementary Materials 2.** The raw Western blot images of CD9, CD63, and FASN.

**Additional file 3: Supplementary Materials 3.** The expression trend of the urinary protein ACLY and PCK1 in different age groups.

**Additional file 4: Supplementary Materials 4.** The correlation between FASN concentrations and RBC counts, AST, ALT ALB, serum Cr, and eGFR levels.

**Additional file 5: Supplementary Materials 5.** The proof of overall similarity index of the manuscript using iThenticate.

**Additional file 6: Supplementary Materials 6.** A certificate of language editing.

## Data Availability

The data in the current study could be available from the corresponding author on reasonable request.

## Abbreviations

ACLY: ATP-citrate synthase
FASN: Fatty acid synthase
PCK1: Phosphoenolpyruvate carboxykinas
TG: Triglyceride
FDR: False discovery rate
HDL-C: High-density lipoprotein cholesterol
LDL-C: Low-density lipoprotein cholesterol
MS: Mass spectrometry
DIA: Data-independent acquisition
TEM: Transmission electron microscopy
NTA: Nanoparticle tracking analysis
PCA: Primary component analysis
*r*: Correlation coefficient
BMI: Body mass index
WBC: White blood cell
RBC: Red blood cell
Hb: Hemoglobin
PLT: Platelet
AST: Aspartate aminotransferase
ALT: Alanine aminotransferase
ALB: Albumin
FBG: Fasting blood glucose
Cr: Creatinine
eGFR: Estimated glomerular filtration rate
CEA: Carcinoembryonic antigen
PSA: Prostate specific antigen
CA 199: Carbohydrate antigen 199
AFP: Alpha-Fetoprotein

## References

[1] Fahy E, Subramaniam S, Brown HA, Glass CK, Merrill AH, Murphy RC, Raetz CR, Russell DW, Seyama Y, Shaw W (2005). A comprehensive classification system for lipids. J Lipid Res.

[2] Fahy E, Subramaniam S, Murphy RC, Nishijima M, Raetz CR, Shimizu T, Spener F, van Meer G, Wakelam MJ, Dennis EA (2009). Update of the LIPID MAPS comprehensive classification system for lipids. J Lipid Res.

[3] Fahy E, Cotter D, Sud M, Subramaniam S (2011). Lipid classification, structures and tools. Biochem Biophys Acta.

[4] Swinnen JV, Brusselmans K, Verhoeven G (2006). Increased lipogenesis in cancer cells: new players, novel targets. Curr Opin Clin Nutr Metab Care.

[5] Lemieux S, Prud'homme D, Bouchard C, Tremblay A, Després JP (1993). Sex differences in the relation of visceral adipose tissue accumulation to total body fatness. Am J Clin Nutr.

[6] Blaak E (2001). Gender differences in fat metabolism. Curr Opin Clin Nutr Metab Care.

[7] DiNicolantonio JJ, Lucan SC, O'Keefe JH (2016). The evidence for saturated fat and for sugar related to coronary heart disease. Prog Cardiovasc Dis.

[8] Chait A, Ginsberg HN, Vaisar T, Heinecke JW, Goldberg IJ, Bornfeldt KE (2020). Remnants of the triglyceride-rich lipoproteins, diabetes, and cardiovascular disease. Diabetes.

[9] Bian X, Liu R, Meng Y, Xing D, Xu D, Lu Z (2021). Lipid metabolism and cancer. J Exp Med.

[10] Menendez JA, Lupu R (2007). Fatty acid synthase and the lipogenic phenotype in cancer pathogenesis. Nat Rev Cancer.

[11] Pavlova NN, Thompson CB (2016). The emerging hallmarks of cancer metabolism. Cell Metab.

[12] Mullen PJ, Yu R, Longo J, Archer MC, Penn LZ (2016). The interplay between cell signalling and the mevalonate pathway in cancer. Nat Rev Cancer.

[13] Brånvall E, Ekberg S, Eloranta S, Wästerlid T, Birmann BM, Smedby KE (2020). Statin use is associated with improved survival in multiple myeloma: a Swedish population-based study of 4315 patients. Am J Hematol.

[14] Lally JSV, Ghoshal S, DePeralta DK, Moaven O, Wei L, Masia R, Erstad DJ, Fujiwara N, Leong V, Houde VP (2019). Inhibition of acetyl-CoA carboxylase by phosphorylation or the inhibitor ND-654 suppresses lipogenesis and hepatocellular carcinoma. Cell Metab.

[15] Snaebjornsson MT, Janaki-Raman S, Schulze A (2020). Greasing the wheels of the cancer machine: the role of lipid metabolism in cancer. Cell Metab.

[16] Pisitkun T, Shen RF, Knepper MA (2004). Identification and proteomic profiling of exosomes in human urine. Proc Natl Acad Sci USA.

[17] Li X, Yang L (2022). Urinary exosomes: emerging therapy delivery tools and biomarkers for urinary system diseases. Biomed Pharmacother.

[18] Erdbrügger U, Blijdorp CJ, Bijnsdorp IV, Borràs FE, Burger D, Bussolati B, Byrd JB, Clayton A, Dear JW, Falcón-Pérez JM (2021). Urinary extracellular vesicles: a position paper by the urine task force of the International Society For Extracellular Vesicles. J Extracell Vesicles.

[19] Street JM, Koritzinsky EH, Glispie DM, Star RA, Yuen PS (2017). Urine exosomes: an emerging trove of biomarkers. Adv Clin Chem.

[20] Merchant ML, Rood IM, Deegens JKJ, Klein JB (2017). Isolation and characterization of urinary extracellular vesicles: implications for biomarker discovery. Nat Rev Nephrol.

[21] Kanehisa M, Furumichi M, Sato Y, Ishiguro-Watanabe M, Tanabe M (2021). KEGG: integrating viruses and cellular organisms. Nucleic Acids Res.

[22] Sung H, Ferlay J, Siegel RL, Laversanne M, Soerjomataram I, Jemal A, Bray F (2021). Global cancer statistics 2020: GLOBOCAN estimates of incidence and mortality worldwide for 36 cancers in 185 countries. CA Cancer J Clin.

[23] Yun JS, Ko SH (2021). Current trends in epidemiology of cardiovascular disease and cardiovascular risk management in type 2 diabetes. Metabolism.

[24] Health effects of dietary risks in 195 countries, 1990–2017: a systematic analysis for the Global Burden of Disease Study 2017. Lancet. 2019;393(10184):1958–1972. 10.1016/S0140-6736(19)30041-8. 10.1016/S0140-6736(19)30041-8 PMC6899507 30954305

[25] Reiner Ž (2017). Hypertriglyceridaemia and risk of coronary artery disease. Nat Rev Cardiol.

[26] Wierzbicki AS, Kim EJ, Esan O, Ramachandran R (2022). Hypertriglyceridaemia: an update. J Clin Pathol.

[27] Blijdorp CJ, Hartjes TA, Wei KY, van Heugten MH, Bovée DM, Budde RPJ, van de Wetering J, Hoenderop JGJ, van Royen ME, Zietse R (2022). Nephron mass determines the excretion rate of urinary extracellular vesicles. J Extracell Vesicles.

[28] Abramson HN (2011). The lipogenesis pathway as a cancer target. J Med Chem.

[29] Couillard C, Bergeron N, Prud'homme D, Bergeron J, Tremblay A, Bouchard C, Mauriège P, Després JP (1999). Gender difference in postprandial lipemia: importance of visceral adipose tissue accumulation. Arterioscler Thromb Vasc Biol.

[30] Cullen P, Schulte H, Assmann G (1997). The Münster Heart Study (PROCAM): total mortality in middle-aged men is increased at low total and LDL cholesterol concentrations in smokers but not in nonsmokers. Circulation.

[31] Freedman DS, Jacobsen SJ, Barboriak JJ, Sobocinski KA, Anderson AJ, Kissebah AH, Sasse EA, Gruchow HW (1990). Body fat distribution and male/female differences in lipids and lipoproteins. Circulation.

[32] Fried SK, Lee MJ, Karastergiou K (2015). Shaping fat distribution: new insights into the molecular determinants of depot- and sex-dependent adipose biology. Obesity.

[33] Karastergiou K, Smith SR, Greenberg AS, Fried SK (2012). Sex differences in human adipose tissues - the biology of pear shape. Biol Sex Differ.

[34] Link JC, Reue K (2017). Genetic basis for sex differences in obesity and lipid metabolism. Annu Rev Nutr.

