# Supplementary material for: Sex differences in FASN protein concentrations in urinary exosomes related to serum triglycerides levels in healthy adults
Source: Lipids Health Dis. 2023 Oct 19;22:176. doi: 10.1186/s12944-023-01936-7 (PMC10588030; doi:10.1186/s12944-023-01936-7)
Supplement: Supplementary file 2 — Additional file 2: Supplementary Materials 2. The raw Western blot images of CD9, CD63, and FASN. [file 12944_2023_1936_MOESM2_ESM.pdf]

Male1 Male2 Male3 Female1 Female2 Female3

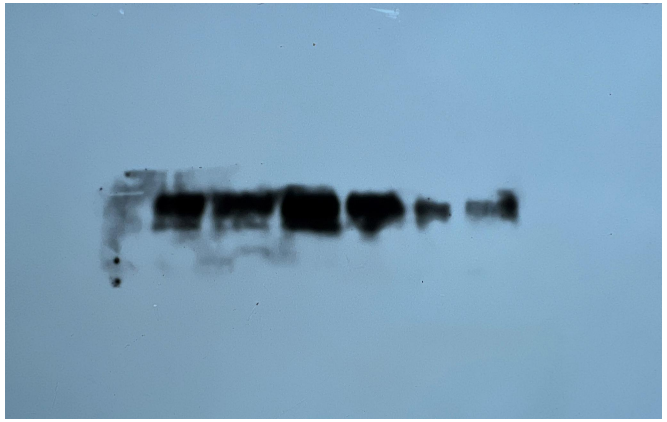

CD63-25kDa

Male1 Male2 Male3 Female1 Female2 Female3

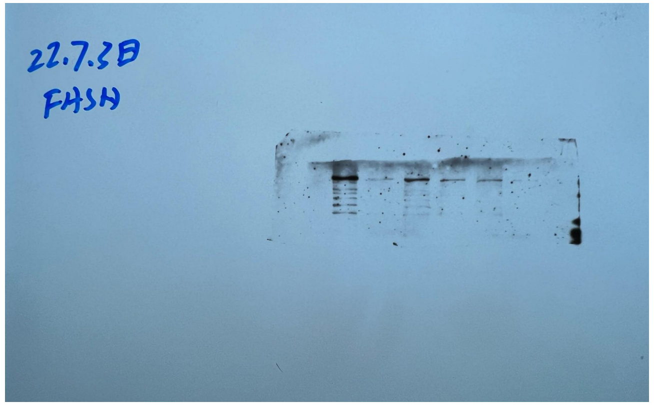

FASN-245kDa

Male7 Male8 Male9 Female7 Female8 Female9

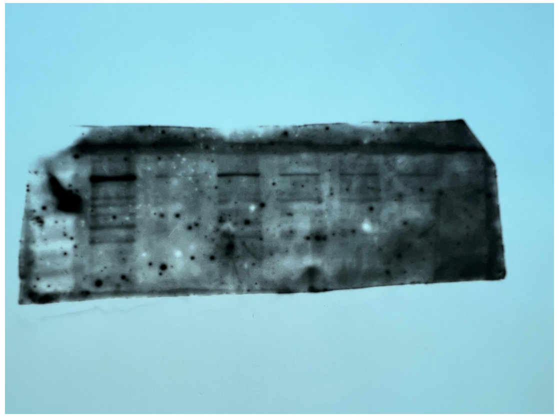

FASN-245kDa

Male1 Male2 Male3 Female1 Female2 Female3

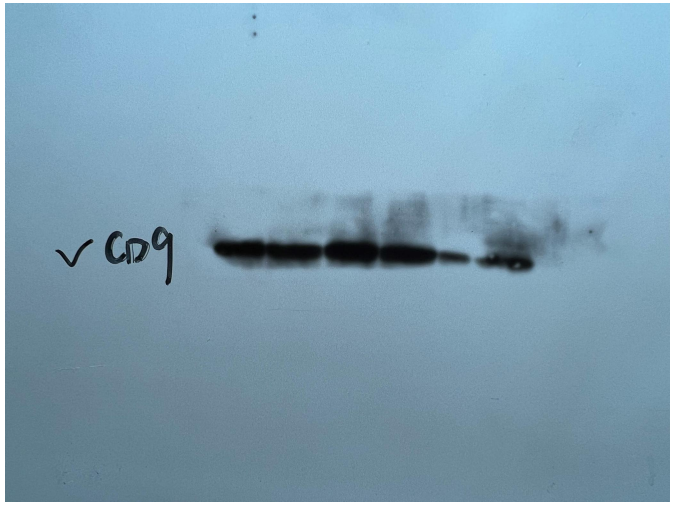

CD9-20kDa

Male4 Male5 Male6 Female4 Female5 Female6

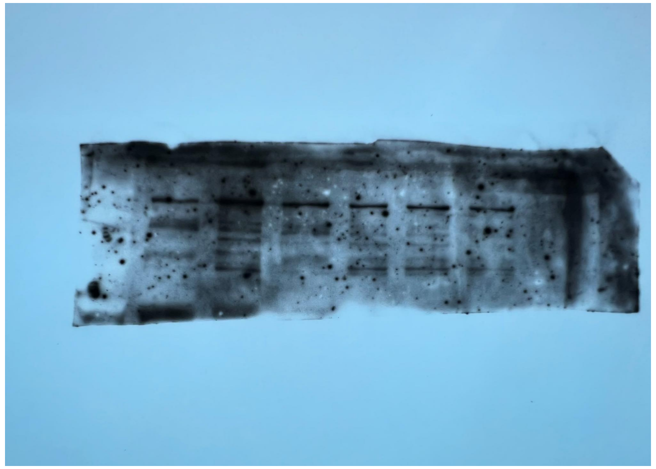

FASN-245kDa

Male10 Male11 Male12 Female10 Female11 Female12

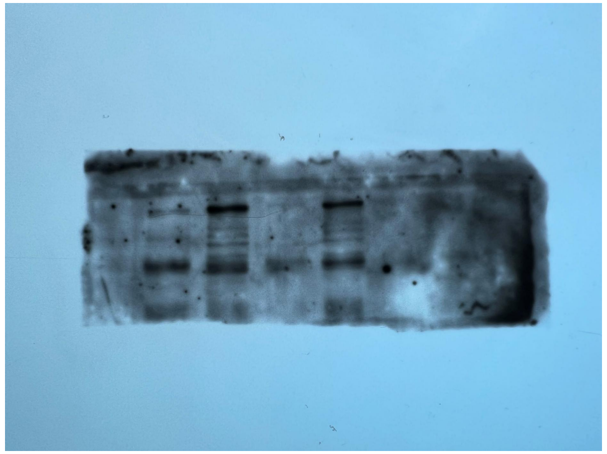

FASN-245kDa
