# Supplementary figures and images for: Sex differences in FASN protein concentrations in urinary exosomes related to serum triglycerides levels in healthy adults
Source: Lipids Health Dis. 2023 Oct 19;22:176. doi: 10.1186/s12944-023-01936-7 (PMC10588030; doi:10.1186/s12944-023-01936-7)

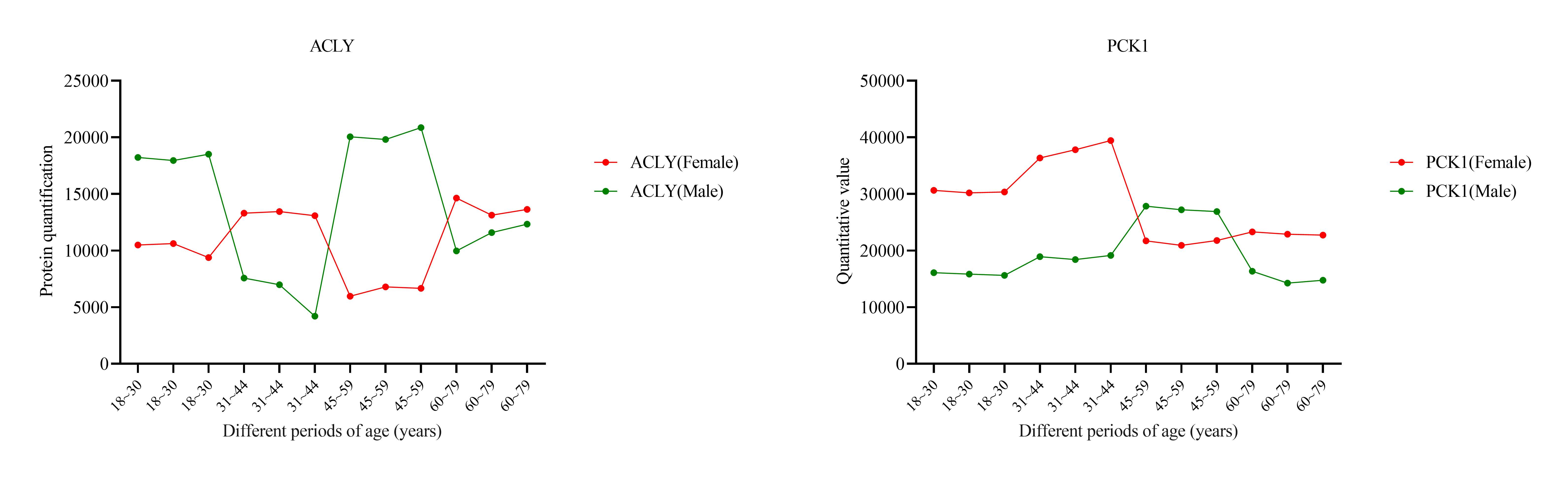

Supplement: Supplementary file 3 — Additional file 3: Supplementary Materials 3. The expression trend of the urinary protein ACLY and PCK1 in different age groups. [file 12944_2023_1936_MOESM3_ESM.tif]

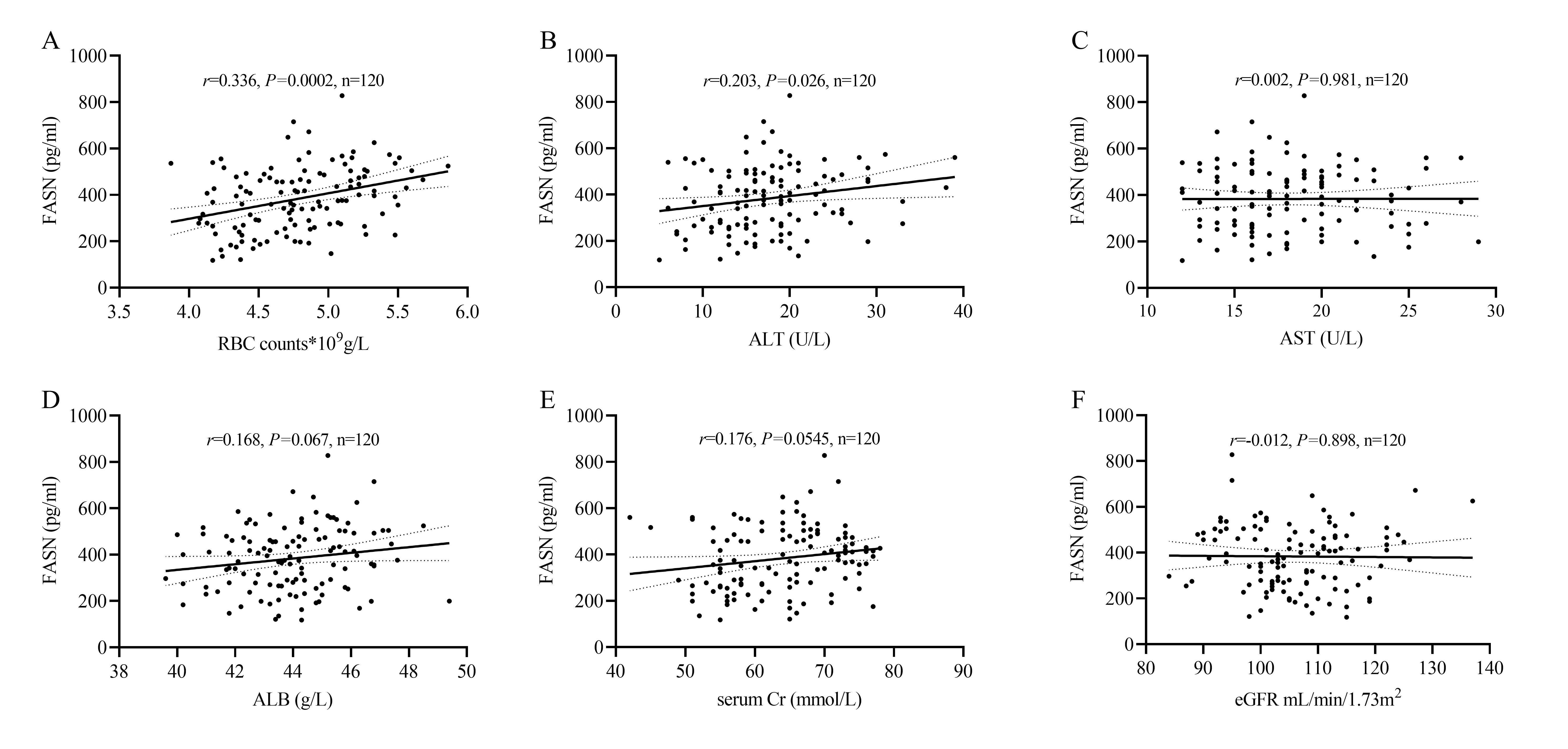

Supplement: Supplementary file 4 — Additional file 4: Supplementary Materials 4. The correlation between FASN concentrations and RBC counts, AST, ALT ALB, serum Cr, and eGFR levels. [file 12944_2023_1936_MOESM4_ESM.tif]
