# Supplementary material for: Sex differences in FASN protein concentrations in urinary exosomes related to serum triglycerides levels in healthy adults
Source: Lipids Health Dis. 2023 Oct 19;22:176. doi: 10.1186/s12944-023-01936-7 (PMC10588030; doi:10.1186/s12944-023-01936-7)
Supplement: Supplementary file 5 — Additional file 5: Supplementary Materials 5. The proof of overall similarity index of the manuscript using iThenticate. [file 12944_2023_1936_MOESM5_ESM.pdf]

20230823015926891544198980726784

WORD COUNT 2881 TIME SUBMITTED 23-AUG-2023 01:59AM
PAPER ID 102175527

Sex differences in FASN protein concentrations in urinary exosomes related to serum triglycerides levels in healthy adults

Abstract

Background: Dysregulation of lipid metabolism is the most prominent metabolic alteration observed in obesity, cancer, and cardiovascular diseases. The present study aimed to explored the detailed changes associated with lipid metabolism in urinary exosome proteins, and evaluate the correlation of urinary exosome proteins with serum lipid biomarkers.

Methods: The key enzymes regulating lipid metabolism in healthy adults were screened using urinary exosome data. Urinary exosomes were isolated from 120 healthy subjects and the expression of urinary proteins was assessed by Western blotting and *ELISA*. The correlation between urinary protein concentrations and the levels of serum lipid biomarkers was analyzed using correlation analysis.

Results: Three urinary exosome proteins, namely fatty acid synthase (FASN), phosphoenolpyruvate carboxykinase (PCK1), and ATP-citrate synthase (ACLY) were identified, and only FASN showed gender differences. Sex differences were also observed in the serum triglyceride (TG) levels. Healthy males had higher FASN levels than females, and a moderate positive correlation was found between FASN concentrations and serum TG levels in healthy males ($r=0.479$, $P<0.001$). FASN concentrations in different age groups were positively correlated with the level of serum TG (18~30 years, $r=0.502$; 31~44 years, $r=0.587$; 45~59 years, $r=0.654$; all $P<0.01$). In addition, FASN concentrations was positively related to the increase in

serum TG levels (range:1.0~1.7mmol/L; r=0.574, P<0.001).

Conclusions: Gender differences were observed in urinary exosome FASN protein levels in healthy adults. FASN protein levels positively correlated with increased serum TG levels. FASN may serve as a novel biomarker to evaluate fatty acid synthesis in the human body.

Background

Lipids are water-insoluble substances, that include phospholipids, sterols, sphingolipids, terpenes, and fatty acids. Lipids based on their constituent molecules can be classified as simple or complex. Simple lipids can be described as those that yield at most two types of products during hydrolysis. Complex lipids are those that produce three or more products after hydrolysis[1, 2]. In addition to providing sufficient energy, lipids are extensively spread in the organelles of cells and involved in various signal transduction pathways[3, 4]. Dysregulation of lipid metabolism, such as elevated serum triglycerides (TG) and cholesterol levels, greatly increases the incidence of cardiovascular diseases and diabetes[5, 6]. Furthermore, numerous studies have demonstrated that lipid metabolism disorders are the most prominent metabolic alteration in cancers, especially breast and cervical cancers[7, 8]. Cancer cells harness lipid metabolism to obtain energy to alter the tumor microenvironment, thereby promoting the survival, proliferation, invasion, and migration of cancer cells. Since homeostasis of lipid metabolism is essential for cancer development, inhibitors of rate-limiting enzymes are promising targets for treatment[9]. Numerous preclinical studies investigating potential inhibitors of a variety of lipid metabolism enzymes for cancer treatment, including inhibitors of cholesterol synthesis[10], statin family drugs[11], and inhibitors of fatty acid synthesis[12, 13], have been reported. The early and timely detection of lipid metabolism disorders is essential for human health.

Urine is the second most commonly used biological fluid in clinical diagnosis, and commonly contains epithelial and blood cells, bacteria, viruses, and exosomes[14].

Urinary exosomes are small circular membranous vesicles with a diameter of 30 ~ 150 nm derived by kidney epithelial cells or urinary tract cells, reflecting the physiological and pathological state of the body in health and diseases[15, 16]. Numerous studies have pointed out that urinary exosomes can serve as a source of novel biomarkers reflecting the physiological and pathophysiological state of the human body[17, 18]. However, while representing noninvasively collected and easily prepared biological samples, the potential role of urinary exosomes in reflecting lipid metabolism in vivo and the correlation of urinary exosome biomarkers with serum lipid metabolism markers remain unknown. Therefore, we hypothesized that biomarkers are present in urinary exosomes and capable of reflecting the status of lipid metabolism.

In this study, rate-limiting enzymes related to the regulation of lipid metabolism, including fatty acid and cholesterol metabolisms were screened using urinary exosome data from healthy subjects. In addition, the expression levels of key rate-limiting enzymes were assessed, and their correlation with serum lipid metabolism biomarkers levels was evaluated.

Methods

Study cohort

Healthy subjects who were first admitted for physical examination at Beijing Shijitan Hospital, were sampled between January 2021 and January 2022. The inclusion criteria for the healthy subjects were as follows: 1) Physical examination showed no tumors, autoimmune diseases, diabetes, cardiovascular diseases, and chronic liver

disease; 2) Laboratory test results were within normal ranges; 3) No acute urinary tract infections were observed. 4) No history of drug abuse. The present study was approved by the Medical Ethics Committee of Beijing Shijitan Hospital (No. sjtkyll-1x-2021(115)) and followed by the principles of the Declaration of Helsinki and. Written consent of all healthy subjects was obtained before being included in this study.

Serum biomarkers measurement

Serum samples were obtained from the peripheral veins of healthy subjects and then centrifuged at a speed of 1500 rpm for 10 minutes. Serum lipid biomarkers were measured using BECKMAN COULTER AU5800 instrument (USA). Complete blood counts were measured using SYSMEX, XE-5000 (Japan). Serum tumor markers levels, including alpha fetoprotein (AFP), carcinoembryonic antigen (CEA), carbohydrate antigen 199 (CA199), and prostate specific antigen (PSA) were measured using the electrochemiluminescence method (Roche Cobas E601, Germany). HbA1c levels were also analyzed in our laboratory (TOSOH G8, Japan).

Urine collection and extraction of urinary exosomes

Fifty mL of urinary samples were obtained from the healthy subjects. Each sample were centrifuged at a speed of 1500 g for 10 min, followed by removing the cells and debris at 10000 g × 30 min. Concentrate processed urine to 10 mL using ultrafiltration tubes. Urinary exosomes were extracted using 35-nm qEV10 iZON size-exclusion chromatography (SEC) columns (H-wayen Biotechnologies, China). The obtained

urinary exosome samples were stored at a temperature of -80°C until usage for further study.

Identification of exosomes derived urine

For transmission electron microscopy (TEM), 10 μl of urinary exosome sample were placed on a dry copper mesh for 10 min. A drop of 2% uranyl acetate was added onto the urinary exosome sample for 1 ~ 3 min. After drying for 10 ~ 15 min, morphology of exosomes under the microscope was acquired (JEM-1200 EX, Japan).

For nanoparticle tracking analysis (NTA), each sample was diluted to an appropriate concentration and directly tested for exosome particle size (ZETA VIEW, PARTICLE METRIX). A typical result of NTA is shown in **Supplementary materials 1**.

Mass spectrometry (MS)

MS proteomic data of enzymes involved on the regulation of lipid metabolism were collected from healthy subjects (Females: C1-F1 and Males: C2-F2; age: 18–79 years). This study focused on the rate-limiting enzymes related to the regulation of lipid metabolism, including fatty acid and cholesterol metabolisms. Simplified workflow of the MS analysis is briefly described as follows. 1 ug of the sample was injected and the liquid quality was tested. Mass spectra were analyzed by an ORBITRAP ECLIPSE mass spectrometer using data-independent acquisition (DIA) mode. The results of MS were obtained in the human database (www.UniProt.org). When fold change > 1.5 and P value < 0.05, it was considered to suggest the presence of differentially expressed. Cluster heat map and primary component analysis (PCA) were produced and visualized using R 4.0.1 version (USA). A detailed workflow of

this study is summarized in **Figure 1.**

Western blotting analysis

The study randomly selected 12 males and 12 females among the 120 healthy subjects. Samples were lysed in RIPA lysis buffer for 30 min. The total urine protein level was measured by BCA Protein Assay Kit (Solarbio, Beijing, China) and selected sample was loaded onto 8% ~ 15% sodium dodecyl sulfate-polyacrylamide gel. Electrophoresis was performed at 60 V to 120 V, followed by transfer to the membrane (Bio-Rad, Hercules, CA, USA). A 5% blocking solution was added, and placed on a rocker for 2 h. Anti-CD9 (Abcam, ab236630), anti-CD63 (Abcam, ab271286), and anti-fatty acid synthase (Proteintech, 10624-2-AP) antibodies were used in suitable ratio of 1:1000~3000, and then overnight incubation at a temperature of 4°C. Next day, washing three times, secondary antibody was added at a ratio of 1:3000 for 2 h. After rewashed three times, chemiluminescent ECL solution (Bio-Rad, cat. #170-5061) was added for exposure. Raw Western blot images are shown in **Supplementary materials 2**.

ELISA analysis of urinary exosome FASN protein level

Samples were lysed in RIPA lysis buffer for 30 min. Total protein was measured using BECKMAN COULTER AU5800 instrument (USA). Concentrations of urinary protein were measured using an *ELISA* kit (Shanghai Enzyme-linked Biotechnology Co., Ltd; lot:202204, Catalog number: YJ84850). Based on the instruction of manufacturer, 50μl of samples were added to each well and then incubated for 30 min at a temperature of 37 °C. After three times of washing the plate, HRP-Conjugate

reagent was added and incubated for 30 min. After washing, 50 µl of chromogen A and B solution were added to each well plate for 10 min at a temperature of 37 °C. Finally, adding stop solution for measuring absorbance. The value of optical density was measured at 450 nm (Thermo Fisher Scientific, USA), with each sample tested three times. The FASN concentrations was calculated based on the standard curve.

Statistical analysis

For normally distributed data, the mean ± SD is presented and compared using Student's t-test. For non-normally distributed continuous variables, Mann–Whitney test were used to analysis. The relationships between the levels of serum lipid metabolism biomarkers and urinary protein concentrations were analyzed by Pearson's correlation or Spearman's rank coefficient. The analysis of all variables was analyzed by GraphPad Prism software (California, USA). Differences in analysis were considered to be significant if $P < 0.05$.

Results

Key lipid metabolism enzymes in urinary exosomes

This study identified three proteins associated with lipid metabolism in urinary exosomes, namely, FASN, ATP-citrate synthase (ACLY), and phosphoenolpyruvate carboxykinase (PCK1). PCA showed that the data for the three urine proteins were reliable (Figure 2A). The clustered heatmap showed a distinction between ages groups for the three urine proteins (Figure 2B). The trends of FASN abundance demonstrated that healthy males had higher levels than females in different age groups (Figure 2C). Sex differences in different age groups for the quantitative value of the urine FASN

protein were observed (all $P < 0.001$; Figure 2D). However, the urine ACLY and PCK1 proteins did not show these trends in gender differences (Supplementary Materials 3). The classical fatty acid synthesis pathway and potential regulatory mechanisms of FASN are summarized in Figure 2E[19].

Clinical characteristics of healthy subjects

Healthy subjects (n = 120) were included in the validation set. The clinical data showed that no difference in age between males and females. Furthermore, healthy males had a higher level of RBC counts, ALT, AST, ALB, and serum creatinine (Cr) levels, and estimated glomerular filtration rate (eGFR) was not ($P < 0.05$; Table 1). Interestingly, sex differences were also observed in the level of serum TG, HDL-C, LDL-C (all $P < 0.01$; Figure 3). Sex differences were not observed in other clinical features.

FASN evaluation by Western blot and ELISA analysis

Representative TEM images of urinary exosomes are shown in Figure 4A (scale bars; a =1 μm, b = 100 nm). Urinary exosome markers, including CD9 and CD63, were identified using Western blotting (Figure 4B). According to the NTA measurements, almost all urinary exosomes are 123.7 nm in size (Supplementary Materials 1). The expression level of FASN was determined by Western blotting. Compared with healthy females, in healthy males there was a trend towards increased levels of urine FASN protein (Figure 4C). Finally, the concentration of the urine FASN protein was analyzed in 120 healthy subjects using ELISA. Significant sex differences were observed in the different age groups (18 ~ 30 years, P=0.047; 31 ~ 44years, P =

0.0013; 45 ~ 59 years, $P = 0.0006$; Figure 4D).

Correlation between urinary protein FASN concentrations and serum TG levels

In healthy participants, a positive correlation was found between FASN concentrations and the level of serum TG ($r=0.447$, $P < 0.05$; Figure 5A). FASN concentrations had a positive correlation with serum TG levels in healthy males ($r = 0.479$, $P < 0.05$; Figure 5C). However, this correlation was not observed in healthy females ($r = 0.223$, $P = 0.086$; Figure 5B).

Meanwhile, FASN concentrations positively correlated with the level of serum TG in different age groups (18 ~ 30 years, $r = 0.502$; 31 ~ 44years, $r = 0.587$; 45 ~ 59 years, $r = 0.654$; all $P < 0.05$; Figure 5 D, E, and F).

Correlation between stratified serum TG levels and urinary protein FASN concentrations

Because the serum TG levels were within normal range, we further divided these healthy subjects into two groups, including low (n = 79; TG range: 0.2 ~ 1mmol/L) and high TG groups (n = 41; TG range: 1.0 ~ 1.7 mmol/L). Interestingly, the trend shown by FASN concentrations was consistent with that shown by serum TG levels (Figure 6A). FASN concentrations positively correlated with high serum TG levels ($r = 0.574$, $P < 0.0001$; Figure 6C). However, positive correlation was not observed in the low serum TG levels group ($r = 0.043$, $P = 0.707$; Figure 6B).

Discussion

Cardiovascular diseases, diabetes, cancer, and obesity are the most common diseases that threaten human health[20, 21]. Homeostasis of the lipid metabolism, particularly

fatty acid synthesis, is crucial for maintaining the functional status of the human body. Fatty acids are the main components of triglycerides, phospholipids, and glycolipids, and provide sufficient energy to support the activities of organisms. Although serum TG level can be used to evaluate overall fatty acid synthesis in the body, it may not serve as an early biomarker for predicting lipid metabolism disorders[22, 23]. Urinary exosomes are emerging sources of biomarkers widely implicated in diseases onset and progression[17]. In this study, FASN, ALCY, and PCK1 proteins were identified in the urinary exosomes of healthy adults. Differences in FASN but not in ACLY and PCK1 proteins concentrations were observed across the sex and age groups. Although serum biomarkers levels were within the normal range, sex differences also existed in RBC counts, AST, ALT ALB, Cr, eGFR, and TG levels. Positive correlations were found between urinary protein FASN concentrations, RBC counts, and ALT levels (Supplementary Materials 4). A recent study has shown that the excretion rate of urinary extracellular vesicles decreases with a decline in nephron mass[24]. In this study, no correlation was found between urinary protein FASN concentration and eGFR. The lack of correlation may be due to the normal renal function in healthy subjects.

The biological function of FASN is to transform excess carbohydrates into fatty acids, which can be esterified into triacylglycerols. Eventually, these stored or used for energy through β-oxidation[8, 25]. This study demonstrated the existence of the urinary exosome FASN protein, which may serve as a non-invasive and stable biomarker to evaluate fatty acid synthesis status. Furthermore, earlier studies have

reported that serum TG levels gradually increase with age[26]. However, serum TG levels in males increase until 45 years and then slightly decrease, whereas TG levels continue to increase with age in females[27]. In this study, FASN concentrations were positively correlated with increased serum TG levels. Freedman et al. found a distinct difference in fat distribution between healthy males and females, and point out that this sex difference was related to differences in lipid levels[28]. Previous studies have demonstrated that females have developed specific mechanisms that facilitate the storage of adipose tissue, whereas males are able to more effectively mobilize stored fat[29, 30]. Differences in the oxidation of base fatty acids, modulation of lipolysis by catecholamines and insulin, and post-prandial fatty acid storage may promote sex differences in regional fat distribution[31]. Jenny and Karen have reported that basic genetic differences are ultimately determined by XX or XY sex chromosome complements between males and females[32].

Metabolism of is a complex and multifactorial process. Urine, an end product of the body's metabolism, is filtered by the kidneys and excreted through the urinary tract. Urinary exosomes may be derived from urinary tract cells or kidney epithelial cells, reflecting the functional state of the human body. Based on these results, the urinary exosome FASN protein may be an end-product excreted by the kidneys that reflects the state of fatty acid metabolism in the body. Sex differences in FASN levels may be due to the beneficial effects of endogenous estrogen in females and different fat storage sites in men and women. A study is being conducted to identify the potential underlying processes for gender differences about FASN levels.

This study has several limitations. First, due to differences in water consumption among healthy individuals, urine samples may contain a lower quantity of urinary exosomes than blood samples. In addition, the lack of serum urinary exosome data corresponding to healthy subjects, sex differences, and correlation between serum exosome FASN protein concentrations and serum TG levels were not evaluated. Finally, as this was a single-center cohort study, the sample size was not large, and the results require a larger sample size for further validation.

Conclusions

This explored the potential clinical applications of urinary exosome FASN protein in healthy adults. Urinary exosome FASN protein showed sex differences in healthy adult and had a positive correlation with increased serum TG levels. This study may provide a novel biomarker for the evaluation and monitoring of fatty acid synthesis, and further prevent the onset of the obesity, cardiovascular diseases, diabetes, and cancer.

20230823015926891544198980726784

ORIGINALITY REPORT

9%
SIMILARITY INDEX

PRIMARY SOURCES

1	Tao Li, Tian ci Liu, Na Liu, Man Zhang. "Changes in urinary exosomal protein CALM1 may serve as an early noninvasive biomarker for diagnosing diabetic kidney disease", Clinica Chimica Acta, 2023<br>Crossref	71 words — 2%
2	www.researchsquare.com<br>Internet	34 words — 1%
3	www.dovepress.com<br>Internet	26 words — 1%
4	Toru Sanai, Ken Okamura, Shuichi Rikitake, Tsuyoshi Takashima, Motoaki Miyazono, Yuji Ikeda, Takanari Kitazono. "Elevated serum thyroglobulin levels as a marker of reversible hypothyroidism in patients with end-stage renal disease due to chronic glomerulonephritis", Cogent Medicine, 2017<br>Crossref	17 words — 1%
5	Dolores Malaspina, Julie Walsh-Messinger, Daniel Antonius, Roberta Dracxler et al. "Parental age effects on odor sensitivity in healthy subjects and schizophrenia patients", American Journal of Medical Genetics Part B: Neuropsychiatric Genetics, 2016<br>Crossref	14 words — < 1%

6 Mutanen, M.. "Comparison of the effects of interesterified butter oil, natural butter oil, rapeseed oil and sunflower oil on postprandial lipoprotein levels in healthy women", Atherosclerosis, 19940915 — 14 words — < 1%
Crossref

7 f6publishing.blob.core.windows.net — 13 words — < 1%
Internet

8 Thuthuzelwa Stempa, Graeme Bradley. "Effect of Sex and Breed on HSPA1A, Blood Stress Indicators and Meat Quality of Lambs", Animals, 2020 — 11 words — < 1%
Crossref

9 koreascience.or.kr — 11 words — < 1%
Internet

10 www.hindawi.com — 11 words — < 1%
Internet

11 article.sapub.org — 10 words — < 1%
Internet

12 www.aging-us.com — 10 words — < 1%
Internet

13 www.frontiersin.org — 10 words — < 1%
Internet

14 coek.info — 8 words — < 1%
Internet

15 journals.lww.com — 8 words — < 1%
Internet

EXCLUDE QUOTES	OFF	EXCLUDE SOURCES	OFF
EXCLUDE BIBLIOGRAPHY	ON	EXCLUDE MATCHES	OFF
