# Supplementary material for: Sex differences in FASN protein concentrations in urinary exosomes related to serum triglycerides levels in healthy adults
Source: Lipids Health Dis. 2023 Oct 19;22:176. doi: 10.1186/s12944-023-01936-7 (PMC10588030; doi:10.1186/s12944-023-01936-7)
Supplement: Supplementary file 6 — Additional file 6: Supplementary Materials 6. A certificate of language editing. [file 12944_2023_1936_MOESM6_ESM.pdf]

editage

Editing Certificate

This document certifies that the manuscript listed below has been edited to ensure language and grammar accuracy and is error free in these aspects. The logical presentation of ideas and the structure of the paper were also checked during the editing process. The edit was performed by professional editors at Editage, a brand of Cactus Communications. The author's core research ideas were not altered in any way during the editing process. The quality of the edit has been guaranteed, with the assumption that our suggested changes have been accepted and the text has not been further altered without the knowledge of our editors.

MANUSCRIPT TITLE

Sex differences in FASN protein concentrations in urinary exosomes related to serum triglycerides levels in healthy adults

AUTHORS

Tao Li

ISSUED ON

August 19, 2023

JOB CODE

XZTMI_1

THE EDITAGE Promise QUALITY GUARANTEE

Vikas Narang

Vikas Narang
Chief Operating Officer - Editage

editage | helping you get published

Since 2002, Editage has helped over 430,000 authors publish around 1.2 million research papers in scholarly journals across over 1000 disciplines through editorial, translation, transcription, and publication support services. Editage is a brand of Cactus Communications (cactusglobal.com), a science communication and technology company.

CACTUS.

GLOBAL :
+1(833) 979-0061 | request@editage.com

CHINA :
400-120-3020或 021-6020-9400 | fabiao@editage.cn

editage.com | editage.co.kr | editage.jp | editage.cn | editage.com.br | editage.com.tw | editage.de
